# Can Color and Motion Information Be Used to Disentangle the Influence of Multiple Light Sources on Gloss Perception?

**DOI:** 10.1177/2041669518803964

**Published:** 2018-10-10

**Authors:** Gunnar Wendt, Franz Faul

**Affiliations:** Institut für Psychologie, Christian-Albrechts-Universität zu Kiel, Germany

**Keywords:** surfaces/materials, light, color, motion, gloss perception, gloss constancy

## Abstract

Previous results suggest that the glossiness of a surface is systematically underestimated when adjacent highlights from different light sources overlap to such an extent that they appear as a single, expanded highlight. Here we investigated how the availability of color- and motion-induced information, which may help to unravel such merged highlights, affects gloss constancy. We used images of computer-generated scenes where a complex 3D object made of glossy material was illuminated by three point light sources, which had varying distances to each other. The point lights were either all achromatic or they differed clearly in their color and the test object was either presented statically or rotating. The subjects had to adjust the smoothness of a match object illuminated by a single achromatic point light so that it appeared to have the same glossiness as the test object. The results show that color information contributes to gloss constancy in this situation: If it was available, the perceived glossiness remained almost invariant with changes in the degree of overlap between the highlights. This suggests that highlights of different color are processed separately. Motion information had no such effect but only led to a general increase in perceived glossiness.

## Introduction

When two surfaces with identical specular reflection properties that differ in shape or are presented under different lighting conditions are compared by a human observer, they will generally be judged as different in glossiness (Fleming, Dror, & Adelson, 2003; Motoyoshi & Matoba, 2012; Nishida & Shinya, 1998; Olkkonen & Brainard, 2010, 2011; Pont & te Pas, 2006; Todd & Norman, 2018; Vangorp, Laurijssen, & Dutré, 2007; Wendt, Faul, Ekroll, & Mausfeld, 2010). Such cases of incomplete gloss constancy are due to the fact that the visual system bases its glossiness estimate on visual cues that are not only affected by the material properties of the surface but also by other factors, such as the object’s shape and the illumination in the scene.

According to physically based reflectance models, the specular reflection of a surface is determined by its microscale structure, more precisely by the distribution of the orientations of so-called microfacets that constitute the surface of the object (Cook & Torrance, 1982; Ngan, Durand, & Matusik, 2005). These tiny surface elements of about the size of the wavelength of light are assumed to reflect the incident light like a perfect mirror. The less the orientations of the microfacets vary, that is, the smoother the microscale structure of the surface, the less the reflected light is scattered around the direction of the mirror reflection.

Specularly reflecting surfaces normally lead to highlights (or generally spoken, to more or less blurry mirror images of the prevailing illumination) within the retinal image of the surface. Properties of these highlights are used by the visual system as cues for the glossiness of the surface: The sharper, smaller, and more intense the highlights, the more glossy a surface generally appears (Beck & Prazdny, 1981; Fleming et al., 2003; Forbus, 1977; Kim, Marlow, & Anderson, 2012; Kim, Tan, & Chowdhury, 2016; Marlow & Anderson, 2013; Marlow, Kim, & Anderson, 2012; Qi, Chantler, Siebert, & Dong, 2014, 2015).

Obviously, there is a close connection between physical reflection characteristics of the surface, properties of highlights, and gloss impressions. However, as already mentioned earlier, the highlight features are also affected by factors that are not related to surface reflection. They act as interfering variables when judging the material of an object. For instance, it was shown that perceived glossiness depends on the shape of an object (Olkkonen & Brainard, 2011; Vangorp, Laurijssen, & Dutré, 2007; Wendt & Faul, 2017). Under otherwise identical conditions, surfaces with higher local curvatures tend to be perceived as glossier than surfaces with lower local curvatures (Nishida & Shinya, 1998; Wendt et al., 2010).

The material appearance of a surface can also be influenced by the geometry of the light field (Fleming et al., 2003; Motoyoshi & Matoba, 2012; Olkkonen & Brainard, 2010; Pont & te Pas, 2006; Todd & Norman, 2018; Wendt & Faul, 2017). In fact, in extreme cases, a polished chrome surface can appear matte (Sève, 1993) and a diffusely reflecting surface can be made to look glossy (Wijntjes & Pont, 2010), depending on the lighting conditions. Simple illuminations like a single point light, constant hemispheric light, or parallel light seem to make material perception more difficult, especially in combination with simple object shapes, such as spheres (Pont & te Pas, 2006; Wendt et al., 2010). There are indications that a higher degree of constancy of material perception can be achieved under complex illuminations that also include indirect light from the environment and that are in agreement with the statistical characteristics of real-world illuminations (Fleming et al., 2003), but even in this case, an influence of object shape on the degree of constancy was found (Olkkonen & Brainard, 2011).

In a recent study (Wendt & Faul, 2017), we have found that even relatively small changes in the geometry of the light field can lead to large changes in perceived glossiness. As the factor responsible for this, we identified the spatial relationship between nearby highlights that are created by different parts of the light field. In this study, we used computer-generated stimuli where a single three-dimensional (3D) object with one of five different complex shapes was illuminated by three point light sources with identical intensities. The geometry of the light field was gradually varied by changing the relative spatial positions of these lights. The results of a matching task showed that the glossiness of an object was systematically underestimated when highlights that were associated with different point lights overlapped in such a way that they merged to a single highlight of larger spatial extent. Under conditions where the highlights of different origin could be distinguished, the objects were perceived as nearly equally glossy as in a situation with a single light source, even if the global highlight patterns differed considerably with respect to the number or the intensity of the highlights. For instance, when all three light sources were located at the same position (which is equivalent to a single point light with triple intensity), the resulting highlight pattern on the surface consisted of much fewer but more intense highlights than in cases where the point lights were spread over a wide spatial range. Nevertheless, surfaces with such different highlight structures elicited almost identical gloss impressions.

Apparently, the visual system erroneously identified a less glossy surface as the cause for the larger size of (merged) highlights, rather than correctly attributing them to a specific structure of the light field (see also te Pas, Pont, Dalmaijer, & Hooge, 2017, who found that a diffuse light source is often confused with two nearby distinct light sources when the light field estimation is based on information from shadows and specular highlights on the surface of an object). This suggests that the discrimination of highlights that are caused by different light sources is critical for an accurate glossiness estimate.

### Aim of the Study

In the present study, we test whether the availability of color information in the form of differently colored highlights and the availability of motion information from object rotation improve gloss constancy performance in situations with overlapping highlights.

Highlights that appear on surfaces of dielectric material approximately have the color of the illumination (Angelopoulou & Poger, 2003; Lee, 1986). This means that the color of the highlights can be manipulated by using differently colored point light sources. We already used such multicolored lighting conditions in the second experiment of our former study (Wendt & Faul, 2017) where the subjects had to adjust the spatial distance between the point lights in the scene (and thus also between the highlights on the surface of the test object) until a certain visual criterion was reached. We found that the distance between the point lights (or between the highlights, respectively) required to make highlights of different color distinguishable from each other was considerably smaller as the distance required when only white lights were used. This finding indicates that the visual system can make use of color information to segregate highlights. However, it is unclear whether this information can also be used by the mechanisms responsible for gloss perception.

**Figure 1.**
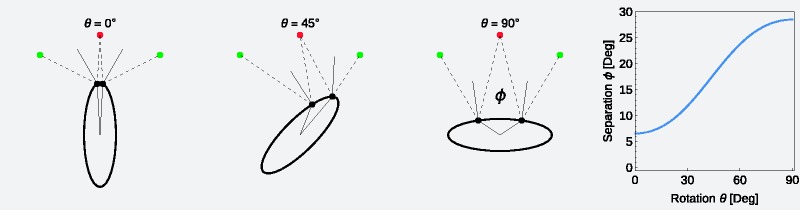
If an object with unequal curvature is rotated relative to an observer, the distance between adjacent highlights belonging to different light sources changes with time. The example shows the top view on an elliptical cylinder rotating around its vertical axis. The object is illuminated by two point light sources (green points). The amount of visual separation (given as visual angle *Φ*) of the two highlights (black points) seen by the observer (red point) depends on the rotation angle *θ*. The plot on the right side shows the systematic change of the separation angle *Φ* with the rotation of the object.

For the same purpose, motion-induced information may be used by the visual system, at least in the case of complex-shaped objects whose surfaces are characterized by a variety of different local curvatures. Figure 1 illustrates in a simple case that object motion, for instance, the rotation of an object around its vertical axis, leads in general to continuous changes in the distance between adjacent highlights.

In contrast to the simple shape used in Figure 1, objects with a complex 3D geometry generally show a much more complex highlight pattern where the highlights may vary in shape, size, and number. Groups of adjacent highlights may show different degrees of overlap at different locations of the same surface due to different local curvatures. The possible contribution of object motion to an improved highlight segregation could be twofold in this context: First, if the object is rotated about an arbitrary axis, the probability is enhanced to capture a surface area where adjacent highlights appear in a degree of overlap that reveals their individual highlight properties to the visual system. This is simply because the object’s surface can be successively scanned from a larger range of views during rotation as in the static case where only a comparatively small section of the surface is visible. Second, during the rotation, one and the same highlight group would pass through different surface locations that generally differ in local curvature. At each location, the spatial structure and the relative distances between the single highlights of a group would generally change. It is possible that such dynamic patterns of changing highlight structures provide sufficient information for the visual system to estimate the properties of individual highlights, even for degrees of overlap that would be below the detection threshold in the static case.

## Experiment 1—The Role of Color and Motion Information in Separating the Influence of Different Light Sources

To test whether the presence of motion and color information in the stimulus affects gloss constancy performance under illumination conditions comprising multiple point lights, we used essentially the same stimuli as in our former study (Wendt & Faul, 2017): They show computer-generated scenes containing a single complex-shaped 3D object of glossy material that is simultaneously illuminated by three different point light sources. The Unity game engine (version 5.6.0f3) was used to present the stimuli.

### Stimuli

**Figure 2.**
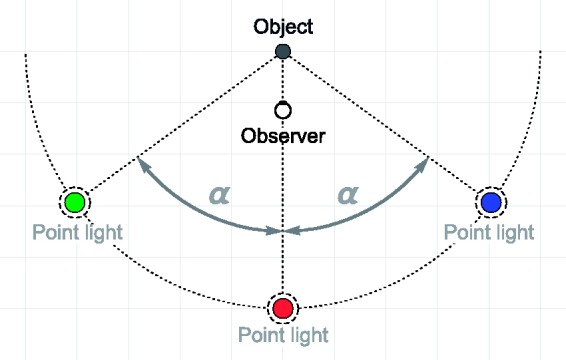
General layout of the test scene (view from top). The test object was located at the center of the scene. One of the three point light sources (red) was always located behind the position of the observer. The positions of the other two point light sources varied during the experiment and were defined by the light spread parameter α which determined the angular distance between the center light (red) and the left (green) or right (blue) light, respectively. All light sources had a constant distance of 5 units to the center of the scene. The two cameras corresponding to the observer’s eyes were located at (−0.03, −1) and (0.03, −1), respectively. Coordinates are given in relative units.

**Figure 3.**
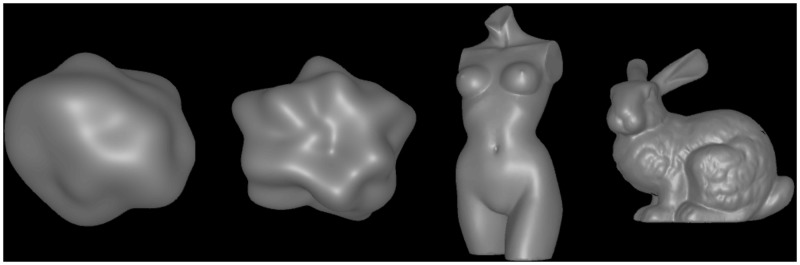
The four test shapes: “blob#1,” “blob#2,” “statue,” and “bunny” (from left to right).

The structure of the test scenes is depicted in Figure 2. All scene elements, that is, the object, the three point light sources, and the two cameras were located in the same horizontal plane. The test object was always placed at the center of the scene. We examined four different object shapes (Figure 3): two blob-like shapes that were generated using the 3D-software blender (version 2.76), the Stanford bunny in a reduced resolution of 55,051 triangles, and a “statue” object that was downloaded from a free database (see https://www.archive3d.net/?a=download&id=c3ba8f71). The construction of the two blob-like shapes “blob#1” and “blob#2” started from an icosphere consisting of 20,480 triangles (after six subdivisions). The “displace” modifier was then applied to create bumpy 3D structures. The parameters of the modifier were as follows: The “strength” parameter was set to 1.0 for both blob shapes, the 3D texture was a cloud texture based on improved Perlin noise with a “size” parameter of 1.0 for “blob#1” and 0.7 for “blob#2”; and the “depth” and the “nabla” parameters were identical for both blobs with values of 0 and 0.03, respectively. Each of the four object meshes was scaled in all spatial directions such that all objects were of similar size: “blob#1” had a height of 4.2 degrees of visual angles (dva), blob#2 one of 4.0 dva, the “statue” 6.3 dva, and “bunny” 4.2 dva.

For the material of the surfaces, the built-in physically based standard shader of Unity was used. The diffuse component (albedo) was set to a constant mid-gray color (rgb = 0.5, 0.5, 0.5). To achieve highlights in the color of the light sources, we set the metallic parameter to 0.0. The smoothness parameter was one of the independent variables. However, it was used in a transformed version, with scaled smoothness = original smoothness^1/1.77^, to obtain an approximately equidistant perceptual scale for the matching task (see Appendix A in Wendt & Faul, 2017). In the following, unless otherwise stated, the term smoothness refers to the parameter scaled in this way.

The test object was always simultaneously illuminated by three point lights. The spatial relationship between these light sources was the second independent variable: All lights were arranged on a virtual circle that had its center at the position of the object and a radius of 5 units (dashed arc in Figure 2). The point light in the middle was always located at a fixed position directly in front of the object (see the red light in Figure 2). The locations of the two remaining point lights were determined by the light spread parameter 0 ≤ α ≤ 1 that stands in a linear relationship to the angle between the positions of the center light and the left (right, respectively) light on the circle. At α = 0, all three point lights are located at the same central position, and at α = 1, the angle between the center and the positions of the left and right point light reaches 90°.

**Figure 4.**
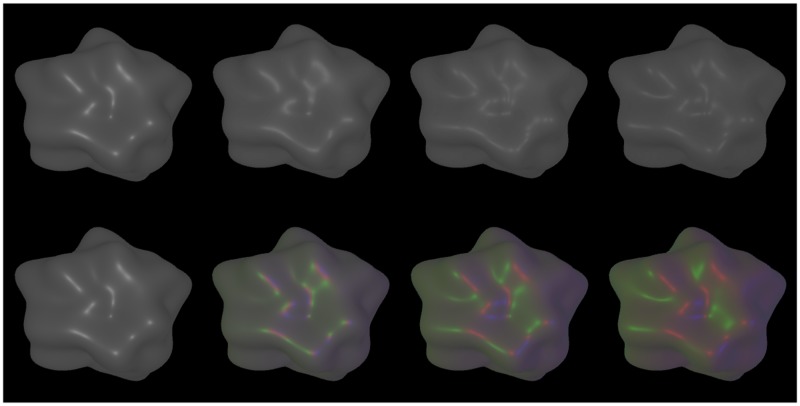
Shape “blob#2” under white (top panel) and multicolored lighting conditions (bottom panel). From left to right, the light spread parameter α is increased from 0.0 to 0.6 in steps of 0.2. All stimuli were rendered using a smoothness value of 0.5. During the experiment, the stimuli were presented stereoscopically.

In the “achromatic” conditions, the point lights had a white color (rgb = 1.0, 1.0, 1.0) and an intensity of 0.5 (for more details, see Wendt & Faul, 2017). In the “chromatic” conditions, colored light sources, all with an intensity of 1.5, were used. In this condition, the central light was red (rgb = 1.0, 0.0, 0.0), the left one green (rgb = 0.0, 1.0, 0.0), and the right one blue (rgb = 0.0, 0.0, 1.0). The effective range of the point lights, that is, the distance at which the light intensity falls to 0, was set to 10 units. The remaining light parameters were the default values of Unity. In addition to the point lights, a constant neutral ambient color (rgb = 0.6, 0.6, 0.6) was used. The other global illumination options, that is, the use of a skybox or a sun source, were disabled. As an example for the stimuli used in Experiment 1, Figure 4 shows shape “blob#2” under the two different lighting conditions “white lights” and “multicolored lights” (rows), each with four different light spread values (columns). As Supplementary Material, we also added a movie, which demonstrates the effects of adding motion and color to the stimuli on perceived glossiness for the entire set of light spread levels used in the experiment (the two monocular half-images of the stimuli are arranged for uncrossed fusion).

The stimuli were presented on a TFT monitor (EIZO CG243W) with a resolution of 1,920 by 1,200 pixels (screen dimensions: 52 cm by 32.5 cm). The CIE1931 color coordinates of the maximum white point were xyY = 0.313, 0.327, 122.57. We used a mirror stereoscope to present the stimuli (ScreenScope). The path length of the light between the monitor and the eyes of the observer was 50 cm. The two monocular half-images of a stimulus were taken from camera positions within the scene that were located at (−0.03, −1.0) for the left eye and at (0.03, −1.0) for the right eye (Figure 2). The starting values for the two camera objects were 0.5 units for the near and 3.0 units for the far clipping plane, and 60° for the field of view. However, since we used the off-axis perspective projection (Kooima, 2008), these values were changed by a script according to this method as soon as the experimental software was started. The two resulting half-images were displayed side-by-side on the monitor without a gap between them. Each had a width of 30% of the monitor width and a height of 50% of the monitor height. The background of the cameras was held in a uniform blueish color (rgb = 0.192, 0.302, 0.475). During the entire experiment, a text field was shown at the center of the screen (between the test and the match stimulus) indicating the already completed and the total number of trials.

### Procedure

Each trial of the Experiment 1 consisted of two different tasks: In the first part, the subjects were asked to interactively adjust the smoothness parameter of a matching object such that its surface appeared as equally glossy as that of the test object. In the second part, the subjects had to judge the actual similarity of the test and the adjusted stimulus with regard to perceived gloss on a rating scale.

In preparation for the experiment, the subjects were carefully instructed in written as well as in verbal form and they completed a small set of example stimuli prior to the experiment while the investigator was present: They were told that in some cases, it might be difficult to achieve a perfect match. They were asked to do nevertheless their best to maximize the similarity between perceived material properties of the test and matching stimulus. The subjects were instructed not to base their similarity judgments on the presence or absence of certain image features, for example, on different highlight colors between the test and the matching stimulus, but to focus exclusively on the strength of perceived glossiness in both stimuli. In particular, it was pointed out that two surfaces may appear equally glossy, even if they are illuminated by light of different color.

During the matching task, the subjects were presented with a test scene in the bottom half of the monitor and a matching scene in the top half. The matching object was always “blob#2” (Figure 3). It always rotated clockwise around its vertical middle axis at a speed of 60°/s and was illuminated by a single point light located at a fixed position in front of the object (the same position as the red point light in Figure 2). The color of this point light was white (rgb = 1.0, 1.0, 1.0) with an intensity of 1.5 and its effective range was the same as that of the point lights used for the test stimulus, that is, 10 units. The subjects used the left and right arrow keys of the keyboard to adjust the glossiness of the matching stimulus.

In each trial, the test object had one of four different shapes (“blob#1,” “blob#2,” “statue,” or “bunny”; see Figure 3). Each test object was presented with three different smoothness values (0.4, 0.5, and 0.6). To test the influence of motion information on gloss constancy performance, the test objects were either presented statically or with a counter-clockwise rotation around their vertical middle axis at a speed of 60°/s. To test the effect of illumination color on gloss constancy, the point lights were all white in half of the trials, and in the other half they were red, green, and blue, respectively (see Stimuli section). As an additional experimental factor, the light spread of the point lights was varied between 0 and 0.6 in steps of 0.1. We chose an upper interval limit of α = 0.6 because in our previous study (Wendt & Faul, 2017) we found that this light spread value was high enough to make the highlights of a group perceptually distinguishable from each other for the stimulus conditions used in the present experiment. We used a total of seven different equally spaced light spread values, since we also found that the data curves exhibit a characteristic U-shape when plotted as a function of light spread, where the position of the minimum depends on the smoothness of the test stimulus. Hence, in order to reveal these curve shapes in detail, a sufficiently dense array of scan points was necessary.

After the subjects completed the matching task by pressing the space bar, they were asked to rate how satisfied they are with the match. To this end, test and adjusted stimulus were still visible and a small text field appeared on the screen (just underneath the text field that displayed the current trial number) with the label “Satisfaction.” The subjects then used the up and down arrow keys on the keyboard to select a number between 0 and 5 to rate the “quality” of the match. A value of 0 meant that the perceived glossiness of the test and the matching stimulus were still vastly different, although the subject did everything possible to achieve the best match. A value of 5, on the other hand, meant a perfect match. After the subjects made their decision, they ended that trial by pressing the space bar again. The next trial started after a short period of 1 second, during which only the bluish background and the trial counter were visible.

Each of the 336 different condition combinations (4 shapes × 3 smoothness levels × 7 lightspread levels × 2 color conditions × 2 motion conditions) was tested 3 times. The entire set of 1,008 test stimuli was presented in random order. The subjects were given as much time as they needed to complete both tasks. They could interrupt a session at any time and resume it at a later date. On average, the subjects needed about 8 hours to complete the entire set, distributed over three to five different sessions.

### Subjects

A total of nine subjects took part in the experiment, all of whom had normal or corrected to normal visual acuity and were tested for color-vision deficiencies with the Ishihara plates (Ishihara, 1967). The subjects either received money or credit points for their participation (with the exception of GW, one of the authors of the present paper). Since the data sets of two subjects showed rather unsystematic data curves and also extraordinarily high variances, they were excluded from any further analysis.

This study was carried out in accordance with the Code of Ethics of the World Medical Association (Declaration of Helsinki) and informed consent was obtained for the experimentation with human subjects.

## Results

### Matching Task

We performed a five-way analysis of variance (ANOVA) on our matching data, taking all varied experimental factors into account, that is, the object shape, the test smoothness value, the light spread, the availability of color information, and the availability of motion information. We found significant main effects for all of these factors as well as a significant first-order interaction for all factor combinations, except for the combination between test smoothness and the availability of color information (see Table A1 in Appendix A for an overview of the results).

**Figure 5.**
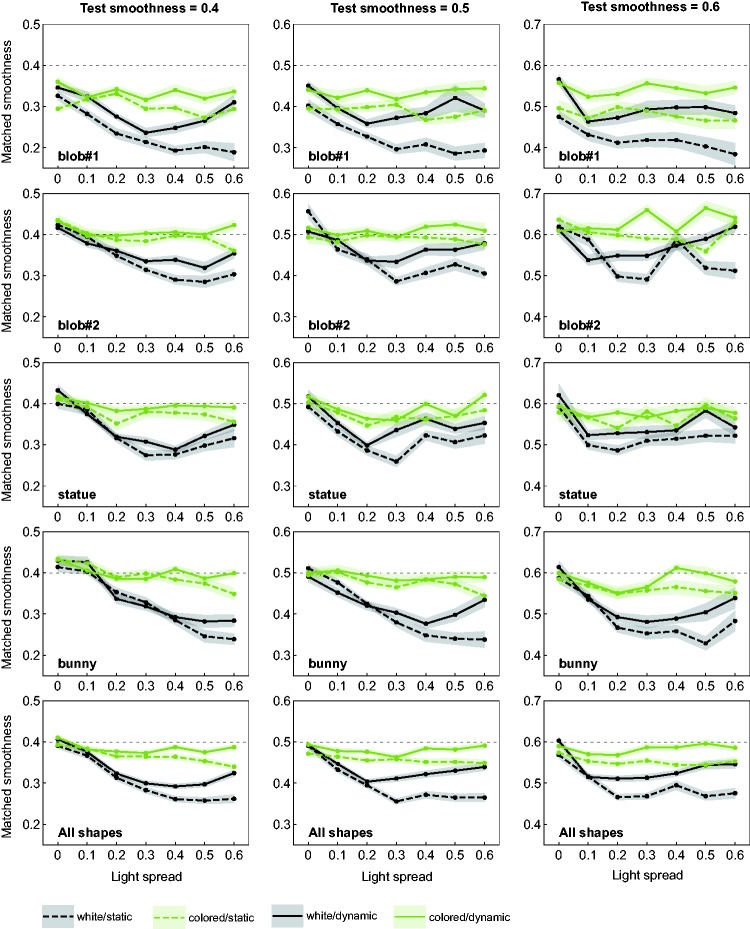
Results of the matching experiment. For each shape condition (top four rows) and test smoothness values (columns), the mean smoothness settings of the match are plotted against the light spread levels. The different curves in each diagram represent the different combinations between the levels of the experimental factors “availability of color information” and “availability of motion information.” The bottom row shows the averaged results across all shape conditions. Transparent areas around each data curve represent ± SEM.

**Figure 6.**
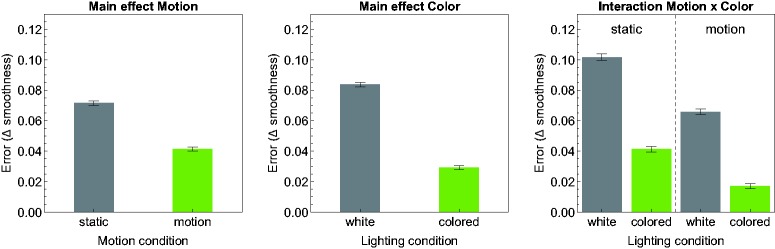
Results of the matching experiment shown in a more condensed form to illustrate the main effects of the two experimental factors “availability of motion information” (left diagram) and “availability of color information” (middle diagram) as well as the interaction effect between them (right diagram). The ordinate (“Error”) refers to the (unsigned) mean Δ smoothness, that is, the deviation of the matched smoothness from the respective test smoothness value, averaged across all subjects and all remaining factors. Error bars represent ± SEM.

Figure 5 gives a detailed overview of the results of the matching task. Each of the top four rows shows the results for one of the different shape conditions. In the bottom row the results are averaged across all four shape conditions. The three diagrams in a row show the results separately for each of the three different test smoothness levels (horizontal dotted line). The data points represent the mean smoothness settings across all seven subjects in dependence of the seven different light spread levels. The data in each diagram are grouped according to the four possible combinations of the levels of the experimental factors “availability of color information” and “availability of motion information” (see the different curves in each diagram). In Figure 6, the results are additionally presented in a more condensed way with a focus on the effects caused by these two latter factors. Here, the mean deviations of the matched smoothness settings from the respective smoothness values of the test stimuli are shown separately for the two factors (left and middle diagram) and for the four different level combinations of these factors (right diagram), averaged across all subjects and all remaining factors.

As a general trend, there seems to be a clear order between these different combinations: Test stimuli with colored lights were generally perceived as glossier than those with white lights (middle diagram in Figure 6) and rotating objects generally appeared glossier than statically presented ones (left diagram in Figure 6). In conditions where the stimuli contained both color and motion information, the mean smoothness settings were close to those values that would be expected under complete gloss constancy. This applies for most of the shape conditions regardless of the light spread level (the exception is “blob#1,” where a systematic underestimation was measured): The colored solid line in each diagram of Figure 5 is almost flat and approaches the reference line of perfect constancy (see also the corresponding bars in the right diagram in Figure 6). Even with static presentation, the settings for stimuli with colored lights seem to be virtually unaffected by the light spread factor (colored dashed line in Figure 5), although they appeared consistently less glossy than their dynamic counterparts.

Without color information available, the data curves generally have a significantly different shape, whereby the smoothness settings are clearly dependent on the light spread. With increasing light spread, the mean settings initially decrease until they reach a local minimum, after which they usually rise again. The position of the local minimum thereby depends on the test smoothness value: The higher the test smoothness, the lower the light spread value at which the local minimum is located.

This characteristic course of the settings was already observed in our previous study (Wendt & Faul, 2017), where all test stimuli were presented statically and under achromatic lighting. As already mentioned in the Introduction section of the present paper, the cause for this nonlinearity is very probably the degree of overlap between nearby highlights. When the highlights of a group appear as a single merged one, the glossiness of the surface is systematically underestimated. This effect depends not only on the spread between the light sources in the scene (which affects the relative distances between the single highlights of a group) but also on the smoothness of the surface (which determines the spatial extent of single highlights). This means that for surfaces with higher microscale smoothness, where the highlights are comparatively small and sharp, the perceived separation into isolated highlights takes place at lower light spread levels. In our former study, we found that after such a split-up into separate highlights the gloss impression almost returned to the original level measured under a light spread parameter value of 0 (i.e. when the surfaces are apparently illuminated by a single point light).

In the present data, this effect seems to be more pronounced under dynamic presentation (black solid lines in Figure 5) than in the static condition (black dashed lines). In the condition that combines white highlights and static presentation, the course of the data curves is more heterogeneous and also seems to be more dependent on the shape of the test object than in the other conditions: While the nonlinearity mentioned earlier can still be recognized in shape condition “statue,” this is not the case with other shapes. There, the curves seem to flatten with increasing light spread or even show a strictly monotonous decrease. However, a common feature of stimuli under this particular condition combination is that they generally produce the weakest gloss impressions over almost the whole range of light spread levels.

### Rating Task

The analysis of the rating data indicates that the subjects were generally very satisfied with the result of their glossiness matches: The mean rating value across all conditions and subjects is 3.62, and only 829 out of 7,056 trials (11.75%) were given a rating value less than 3, with 494 of these 829 lower rated stimuli coming from a single subject. A five-way ANOVA that we performed on the rating data revealed significant main effects for all factors: For “shape,” *F*(3,6720) = 166.38, *p* < .001; for “test smoothness,” *F*(2,6720) = 17.2, *p* < .001; for “light spread,” *F*(6,6720) = 91.8, *p* < .001; for “availability of color information,” *F*(1,6720) = 71.97, *p* < .001; and for “availability of motion information,” *F*(1,6720) = 272.19, *p* < .001. Some noteworthy findings from this analysis are that the satisfaction with the glossiness matches is higher for rotated objects than for statically presented ones and higher for white highlights than for colored highlights, and that the rating values decrease monotonically with the light spread level.

**Figure 7.**
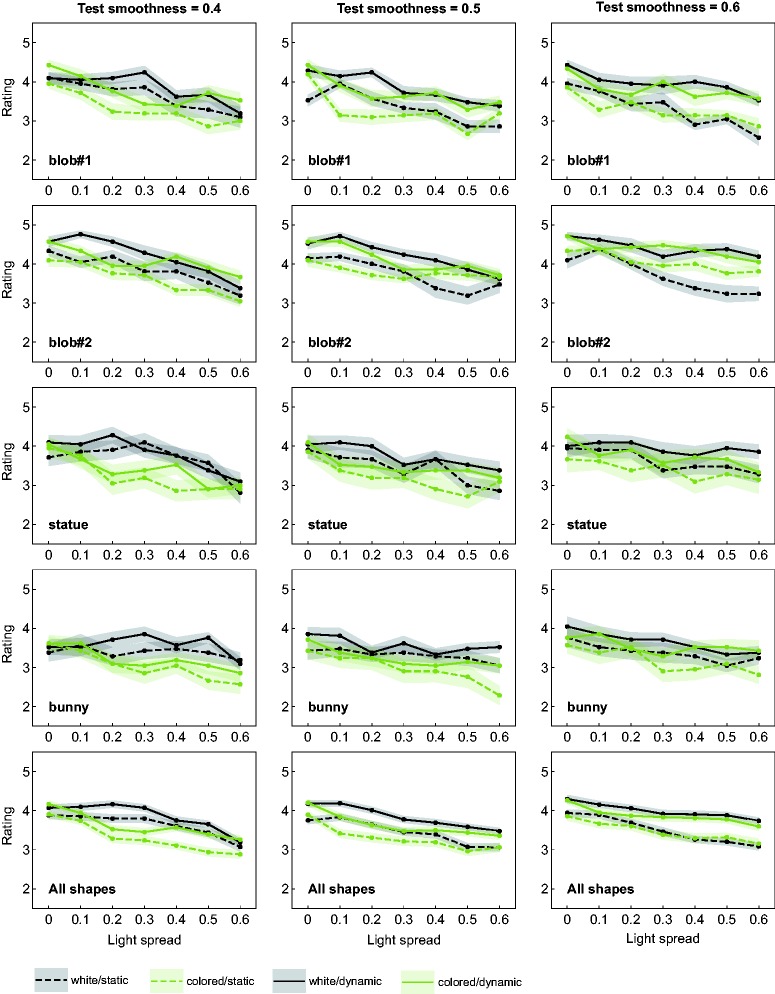
Mean ratings of the goodness of match. For each shape condition (top four rows) and test smoothness values (columns, from left to right: smoothness = 0.4, 0.5, and 0.6), the mean ratings are plotted against the light spread levels. The four curves in each diagram represent different combinations between the levels of the factors “availability of color information” and “availability of motion information.” The bottom row shows the averaged results over all shape conditions. Transparent areas around each data curve represent ± SEM.

A more detailed analysis provides indications of further regularities and interactions beyond these general trends: Figure 7 shows the results of the rating task. The general layout is the same as in Figure 5, but here the single data points give the mean rating values across all subjects in dependence of the light spread value. In the diagrams at the bottom, the data are averaged across all shape conditions. Although the curves differ only slightly in absolute rating values, there seems to be a pattern as to their order: For the two highest test smoothness values of 0.5 and 0.6 (middle and right diagrams), the two curves belonging to conditions with motion information (solid lines) seem to group together. The same seems to apply to the other two types of curves as well just at a comparatively lower level of the rating scale (dashed lines).

For the lowest test smoothness value of 0.4 (left diagram), the availability of color information seems to be the grouping factor, rather than the availability of motion information. As a trend, stimuli with white highlights were rated slightly higher than those with colored highlights, regardless of whether they were presented statically or dynamically. Comparing the top four rows in Figure 7 with each other, this pattern appears to be reasonably stable across the different shape conditions.

## Discussion

The most prominent finding with respect to the matching task is that the availability of color information in the stimulus seems to be sufficient to nearly eliminate the influence of the light spread on the perceived glossiness of the test surfaces: Regardless of whether the objects were presented dynamically or statically—when the highlights of a group were produced by light sources of different color, the perceived glossiness of the stimuli remained almost unchanged when the degree of overlap between the highlights (or the spread of the light sources, respectively) changed. Remaining deviations from the test smoothness value, which seem especially pronounced under shape condition “blob#1” (see top row in Figure 5), seem to be mainly due to differences in the 3D geometry between the match and the test stimulus (see also Wendt & Faul, 2017). In contrast, overlapping highlights with identical colors have a significant effect on perceived glossiness, making a surface systematically appear less glossy (see also the middle diagram in Figure 6).

The results provide strong evidence that color information can help to disentangle the influences of different light sources on the highlight pattern. However, due to the design of the experiment, it is not clear on which aspect of the color information the visual system relies to this end. Both chromatic or luminance differences between highlights could have played the major role: In our multicolor condition, the lights were scaled versions of the three monitor primaries. To obtain an achromatic color when mixed together (i.e., when located at the same point in space), the scaling factor was the same in all three lights. The relative luminances of these lights, however, were very different (the red light contributed about 21% to the luminance of the achromatic mixture, the green one about 73%, and the blue light only 6%). This means that the highlight groups belonging to different light sources systematically differed not only in chromaticity but also in intensity.

## Experiment 2—The Role of Systematic Intensity Differences Between Highlight Groups

In this experiment, we examined the relative role of chromaticity and luminance played in the color effect observed in Experiment 1, by retaining only the intensity aspect and dropping the chromaticity information. To this end, three achromatic light sources were used whose intensities equaled those of the lights in the multicolor lighting condition in Experiment 1.

### Procedure

The color of all three point lights in the test scene was set to rgb = (1.0, 1.0, 1.0). However, contrary to the white light condition of Experiment 1, where all lights had the same intensity weight of 0.5, we used different weights for the lights that met two conditions: First, on a luminance basis, the three single lights had to contribute the same relative amounts to their mixture as the red, green, and blue light in our multicolor condition, namely, 21.16%, 72.51%, and 6.33% of the total luminance, respectively. Second, the final intensity weights that were used to scale the rgb values of the three lights had to sum up to a total of 1.5, as it was the case in all lighting conditions in the main experiment. This led to intensity weights of 0.453, 0.795, and 0.253 for the center, left, and right light source, respectively (see Figure 2).

Since, in this experiment, we were not specifically interested in effects due to different 3D geometries, we only tested three of the four shape conditions used in the main experiment, namely, the shapes “blob#2,” “statue,” and “bunny.” Except for the change in the lighting conditions and the reduced set of shapes, Experiment 2 was identical to Experiment 1.

### Subjects and Sample Size

Four of the subjects who also participated in the main experiment were tested, including one of the authors (GW). Again, each of the 126 stimulus conditions (3 shape conditions × 7 light spread conditions × 3 test smoothness values × 2 levels for the factor “availability of motion information”) was tested 3 times, so that in total 378 trials had to be completed which were presented in random order.

## Results

**Figure 8.**
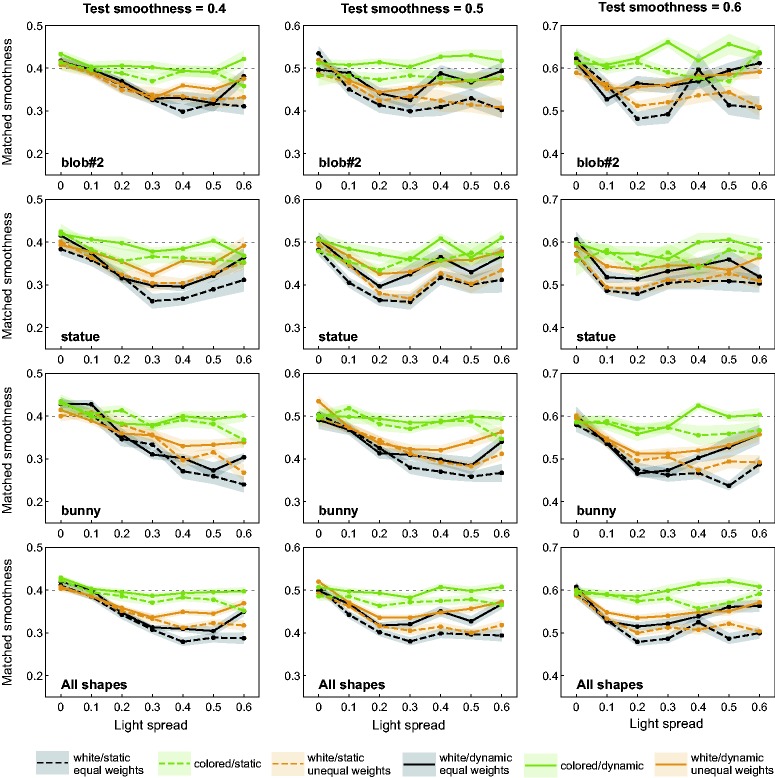
Results of the additional matching experiment with achromatic point lights that differ in luminance (orange curves) in comparison to the respective conditions of the main experiment (black curves for achromatic lights with identical luminances and green curves for differently colored lights), each for dynamic and static presentation (solid and dashed curves, respectively). Each data point represents the mean smoothness settings of the match averaged across those four subjects who participated in both experiments. For each of the three shape conditions (rows) and test smoothness values (columns, see the horizontal reference lines), the mean settings are plotted against the light spread levels. The bottom row shows the data averaged across all shape conditions. Transparent areas around each data curve represent ± SEM.

Figure 8 compares the results of Experiment 2 with the respective data from Experiment 1 for the four subjects who participated in both experiments. As in Figure 5, the mean smoothness settings across all subjects are plotted against the light spread levels for each of the different combinations between the availability of color and motion information (see the different curves in each diagram). The two additional condition combinations that refer to an achromatic illumination with different intensities for the three point light sources are shown as orange curves in each diagram (the solid orange curve belongs to dynamic, the dashed to static presentation).

The results suggest that there is a slight improvement of constancy performance when the white lights differ systematically in their luminance instead of having identical intensities (compare the orange with the corresponding black curves in Figure 8). However, in comparison to the cases where the lights also differed in hue (green curves), the smoothness settings are still considerably affected by the light spread variable, that is, by the degree of overlap between nearby highlights.

Compared with the white light condition investigated in Experiment 1, in which all light sources had identical intensities, a systematic difference in the intensity of the highlight groups belonging to different light sources led to a slightly enhanced gloss impression and slightly improved gloss constancy. However, compared with the multicolor condition, perceived glossiness still showed a strong dependence on the light spread, indicating that it was the chromatic information in the multicolor condition that was mainly responsible for the strong increase in gloss constancy.

Clearly, this effect will also depend on the strength of the chromatic differences between the light sources. In this respect nearly optimal conditions were realized in the experiments: Since we used the colors of the monitor primaries for the three point lights, the hue differences were maximal for the display device used in the experiments. It is to be expected that the effect of light source chromaticity on gloss constancy vanishes if the chromatic differences between light sources fall below a certain threshold.

## General Discussion

In a previous study (Wendt & Faul, 2017), we had examined scenes with multiple light sources, in which adjacent highlights caused by different light sources can overlap to give the impression of an enlarged single highlight. The results of this study suggest that under the given conditions, it was not possible to identify the true cause of such magnified highlights, namely, the interaction of multiple light sources, but that they were misinterpreted as an indication of reduced surface gloss.

The main aim of the present work was to investigate whether object motion and differences in light source color provide enough information to disentangle merged highlights and thus allow a more accurate estimate of the material properties of glossy surfaces. The results of Experiment 1 indicate that color information alone was indeed sufficient to almost completely counteract the effects of merged highlights on gloss perception, whereas object motion had no such effect. Furthermore, the results of Experiment 2 show that the effect of light source color observed in Experiment 1 can mainly be attributed to the *chromaticity* of the light source.

The findings in Wendt and Faul (2017) and the present study give rise to a more refined theoretical picture of how the visual system interprets highlights in the retinal input. We first discuss this with respect to the role of color and motion and then outline how the methods used in the present study may be used as a general paradigm to investigate grouping processes in gloss perception.

### The Role of Highlight Color

**Figure 9.**
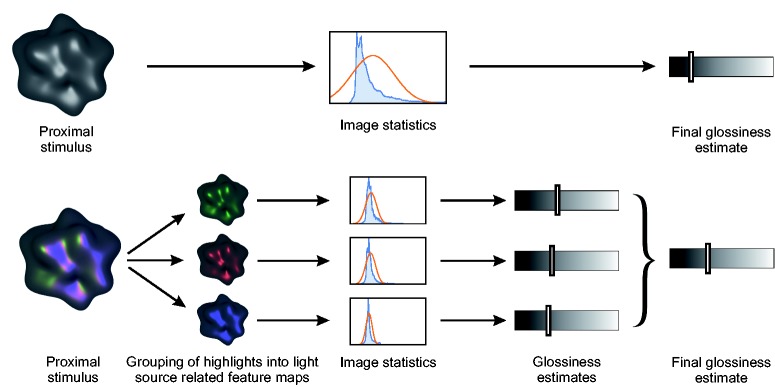
Current approaches to gloss perception consider statistics of the total highlight pattern (top panel). The results of Experiment 1 suggest that under favorable conditions the global highlight pattern may first be split up into subpatterns belonging to different light sources, which are first analyzed separately. Individual estimates made in this step are then integrated into an overall gloss estimate. Specifically, we found evidence that light source chromaticity can be used as a grouping criterion, but it seems plausible that there exist additional grouping features (bottom panel).

The present results suggest that color information is used by the visual system to analyze and demerge the complex highlight pattern and possibly even to decompose it into its causal components: A plausible interpretation of our findings is that the highlights are perceptually grouped according to their different colors (using the Gestalt principle of similarity, see Brooks, 2015), which leads to three separate highlight maps, each associated with one of the three light sources. These highlight maps could then be used to infer independent glossiness estimates, which eventually would be integrated in some way into a combined estimate. The traditional and the alternative view are illustrated in Figure 9.

It has already been found that color information can improve gloss constancy performance (Wendt et al., 2010). However, the way in which color information contributed to an improvement of gloss constancy in this previous study is completely different from the one just described: In Wendt et al. (2010), computer-generated objects with a simulated dielectric material were used that were illuminated by a single achromatic point light source. To create a chromatic difference to the achromatic highlights, a yellow surface color was chosen. The resulting hue transitions can be used to better determine the spatial extent of the highlights, which in turn could improve the estimate of the glossiness of the surface (see also Tominaga & Tanaka, 2000) and increase the degree of perceived glossiness (Hanada, 2012). In pioneering work in computer vision, Shafer and coworkers presented a *dichromatic reflection model* that can be used to isolate highlights from surface reflection in a color image (Klinker, Shafer, & Kanade, 1988, 1990; Shafer, 1985). In the present study, however, we used surfaces with an achromatic diffuse component under multicolored illumination. Hence, the color of the lights affected both the diffusely reflected light and the color of the highlight equally (see also Lee & Smithson, 2017): As can be seen in the bottom panel of Figure 4, the colored highlights are generally surrounded by areas of the diffusely reflecting surface that have the same hue. This can most easily be seen at higher light spread levels where the saturation is enhanced due to the smaller overlap of differently colored highlights. This means that in this case color information does not help to discern highlights from the diffusely reflecting parts.

The finding that chromatic information influences the interpretation of the highlight pattern has far-reaching consequences, because it implies that theoretical approaches to gloss perception, which focus solely on the luminance of highlights, are at least incomplete (see also Hanada, 2012). The assumption that the luminance channel contains most of the information relevant for gloss perception is often made tacitly. For instance, Qi et al. (2014, 2015) proposed a model which aims to predict the glossiness judgments of an observer by a linear combination of a set of global image statistics, such as the average luminance or size of the highlights, or the relative proportion of the surface that is covered with highlights (“percentage highlight area”). A first step necessary for the calculation of these image statistics is to determine the highlight areas in the stimulus. To this end, these authors propose a luminance-based criterion (specifically, each pixel in the stimulus exceeding a certain luminance threshold is considered as part of the highlight pattern). Since, in our present study, these image statistics would be identical for both the multicolor condition of Experiment 1 and the achromatic lighting condition with different weights for the three-point lights realized in Experiment 2, such a mechanism would be unable to predict the differences in perceived glossiness that we actually found between these conditions. Similarly, approaches that relate to relatively low-level global image statistics, like the skewness of the luminance histogram (Motoyoshi, Nishida, Sharan, & Adelson, 2007), would also be incomplete, because to be in line with the present results it would be necessary to assume the computation of separate histograms for each light source.

Incorporating the assumption of separate highlight maps into the model of Qi et al. (2014, 2015) leads to a correct prediction of our results: If the algorithm proposed by Qi and coworkers is applied to each of the separate highlight maps, there is at least one image statistic, the “percentage highlight area,” that would have different values in the multicolor and the achromatic lighting condition. This image statistic has been found to be a strong predictor for the glossiness of a surface in several studies (Qi et al., 2014, 2015; Wendt & Faul, 2017; see also Marlow & Anderson, 2013; Marlow et al., 2012, who used a similar but more general image cue in their model). In our white light condition, the highlight pattern would be extracted in its entirety (due to a lack of grouping features), which would result into a comparatively high value for the “percentage highlight area.” In the multicolor condition, however, the highlight pattern would be separated into three different highlight maps, where each of these maps would have a much lower value for the “percentage highlight area.” In our previous study (Wendt & Faul, 2017), we found that this image statistic is negatively correlated with perceived glossiness, that is, a surface appeared the glossier the smaller the relative size of the highlight area. Hence, if we assume that a glossiness estimate is made for each of the highlight maps (which in a further step would then be combined somehow), this could explain why in the present study our multicolored stimuli generally appeared considerably more glossy than the achromatic ones.

Other highlight features that have also been found to be correlated with perceived glossiness, such as their sharpness or the contrast between the highlight and the diffusely reflecting areas of the surface (Marlow & Anderson, 2013; Marlow et al., 2012), may play a different role in this context. Generally, it is obvious that highlight contrast is strongly correlated with the image statistic “percentage highlight area,” at least under the lighting conditions realized in our present experiments: The more the highlights of a group overlap, the smaller the “percentage highlight area” and the stronger the intensity contrast of the merged highlights. Both statistics, that is, small highlight area and high intensity contrast would consistently indicate a higher degree of glossiness. In the separated highlight maps that would result from grouping processes, however, these two highlight features might actually play antagonistic roles: As we have shown earlier, the statistic “percentage highlight area” would be reduced in the single highlight maps, indicating an even higher degree of glossiness as in the original image. However, at the same time, the contrast in the single highlight maps would generally decrease as well when the highlights appear in their isolated form—which, conversely, could be taken as a cue for weaker glossiness. At present, it is not known how the visual system resolves such conflicts between different image cues. Our present results may be an indication that the image statistic “percentage highlight area” has a stronger weight in cue integration than “highlight contrast.”

### The Role of Object Motion

In our study, the influence of object motion on gloss perception in scenes with multiple light sources seems to be of a completely different nature than the influence of light source color. Our results show that in general dynamic stimuli were perceived as glossier than statically presented ones. This finding was to be expected and confirms previous studies which also reported positive effects of a dynamic presentation on the strength of perceived gloss (Doerschner et al., 2011; Hartung & Kersten, 2002; Sakano & Ando, 2010; Wendt et al., 2010). Apart from such a general increase in perceived glossiness, the availability of motion information apparently was not used to reduce the influence of the light spread variable on perceived glossiness: Although we found a significant interaction between the factor “availability of motion information” and the light spread variable (see Table A1 in Appendix A), the graphs in Figure 5 seem to suggest that in general the addition of motion information only led to an upward shift of the curves but did not flatten their shapes. This indicates that this kind of information did not contribute to the segregation of merged highlight groups.

**Figure 10.**
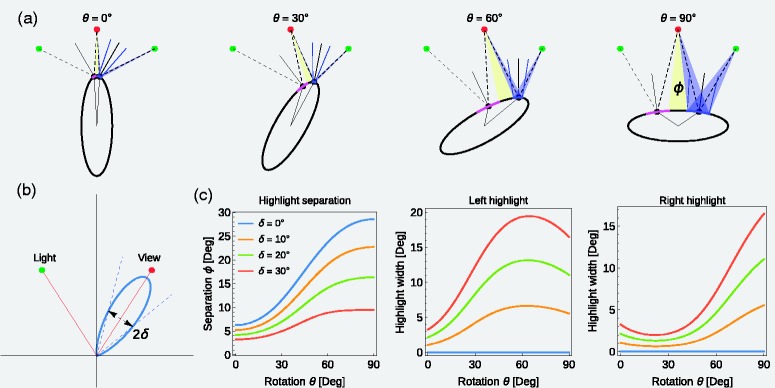
Changes in size and separation of two highlights on a glossy elliptical surface with object motion. (a) Four snapshots of a top view on a rotating elliptical cylinder. Both the width (magenta and blue arc) and the separation between the highlights (yellow region) changes systematically with rotation angle *θ*. The blue-shaded area shows for the right highlight, how its width is computed. (b) In the computation, the specular lobe (according to the Phong-Model; Phong, 1975) of a glossy surface is approximated by a cone with opening angle 2δ, which intersects the lobe at a relative intensity of 0.5. (c) Highlight separation and the width of the left and right highlight depending on rotation angle and roughness parameter δ for the situation depicted in (a)*.*

Given the relationship between object motion and the degree of separation between adjacent highlights demonstrated in Figure 1 and the fact that we used complex shapes with a wide range of curvatures, we were somewhat surprised by this result. On closer inspection of this regularity, however, several additional influences became apparent, which weaken and in part counteract the positive effect of object motion on highlight separation. A first observation is that the range of highlight separations generated by object motion depends on the range of surface curvatures on the object’s surface. For example, the smaller the deviations of a blob object from a spherical shape, the less the distance between adjacent highlights belonging to different light sources varies during object motion. A second counteracting factor is due to the fact that in general the size of a highlight produced by a point light source also depends on the curvature of the surface. As can be seen in Figure 10, the widths of the highlights increase with decreasing curvature. This has two consequences: First, it counteracts in part the increase in highlight separation, because the opposite edges of adjacent highlights are closer to each other than their center. Second, although the separation is nevertheless somewhat increased, the concomitant enlargement of each individual highlight may indicate a decline in surface smoothness and thus a reduced glossiness. A third factor that could make it difficult to take advantage of varying highlight separations during object motion in glossiness judgments can be appreciated in Figure 4: On surfaces of complex shape, adjacent highlights caused by different light sources often differ in shape and extent and (due to partial shadowing) can even disappear completely at some locations. For this reason, it is often not evident from the motion pattern alone, to which light source a highlight belongs. Without additional information, it could therefore be difficult to use information generated by object motion to split the highlight pattern in light source specific maps.

The negative result in the present experiment should therefore not be taken as unequivocal evidence against a positive effect of object motion on gloss constancy in situations with multiple light sources. The complex interplay of different factors, which is partly outlined earlier, requires a more specific investigation that controls or systematically varies such influences. It is quite possible that motion-related information can be used in more favorable conditions than were realized in the present experiment.

### Other Grouping Features

Besides color as a grouping factor, one can imagine further highlight features that could be used by the visual system to decompose complex highlight patterns into separate highlight maps, belonging to different light sources.

For instance, if the light sources that are present in the scene differ in their spatial properties, such as their extension or shape, this would also have a systematic effect on the spatial properties of the highlights in the image (van Assen, Wijntjes, & Pont, 2016). Such systematic differences in the forms of local highlights could potentially be used to group similar highlights together and in this way to decompose the global highlight pattern into light source-specific subpatterns (Brooks, 2015). Similar effects are to be expected in dynamic lighting conditions, for example, when the individual light sources move around the object on different paths or at different speeds. The resulting highlight pattern would show divergent motion patterns which potentially could provide sufficient information to allow an assignment of local highlights to different light sources that can be analyzed separately. In such cases, grouping based on the Gestalt principle of common fate could be used (Ahlström, 1995; Börjesson & Ahlström, 1993).

Clearly, such specific hypotheses need to be tested empirically. To this end, the approach presented in the present study could provide a useful experimental paradigm. The main change that is necessary to adapt the paradigm to other potential grouping factors would be to replace the role that color and motion played in the present study with the corresponding cue. Whether the tested cue actually contributes to a decomposition of the global highlight map, can then be judged by its effect on the glossiness settings depending on highlight separation. The result pattern observed in the present study for color and motion can be considered as typical for an effective and an ineffective grouping cue, respectively.

### Interindividual Differences

**Figure B1.**
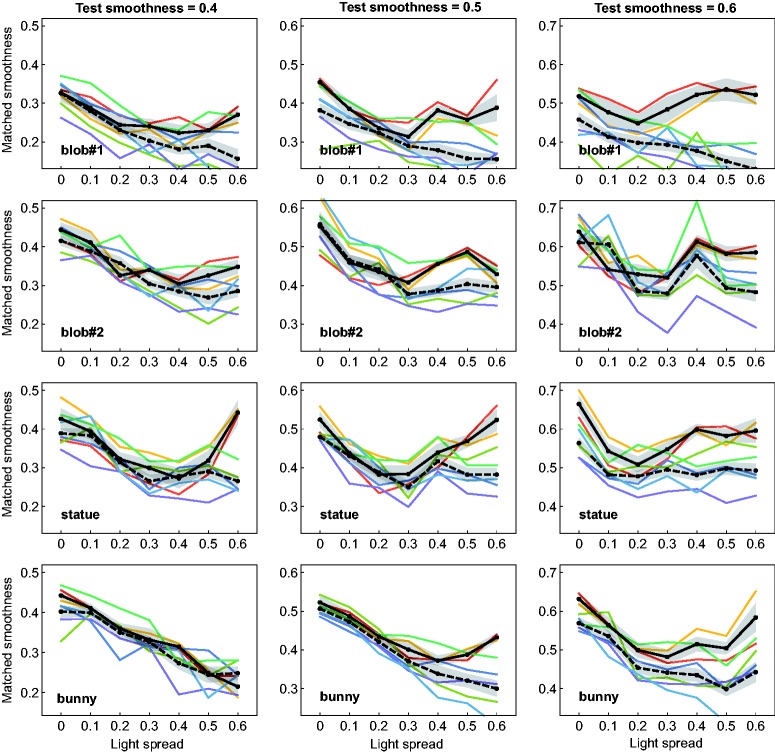
Results of the matching experiment for the condition combination static/white lights, separately shown for the two different groups of subjects who differ in their data trends. The black solid lines show the results of the group (with *n* = 2; see the red and the yellow curves for the two subjects) that roughly produced U-shaped curves in almost all cases (except for the “bunny” shape condition under test smoothness value 0.4, see the bottom left diagram), that is, the same trend as it was found in our previous study for this particular condition combination. Dashed black curves refer to the group (*n* = 5; for the individual subjects of this group see the curves colored in the blue–green spectrum) with clearly different trends. For each shape condition (rows) and test smoothness value (columns, see the horizontal reference lines), the mean smoothness settings of the match are plotted against the light spread levels. Transparent areas around each group curve (black lines) represent ± SEM.

There is one aspect in our data that seems to be at odds with one of our previous findings (Wendt & Faul, 2017): For those stimulus conditions that we already examined in our former study, that is, the static stimuli under white lights (see the dashed black lines in Figure 5), we obtained data curves that show some differences between the two studies, at least when averaged across all subjects. The general trend in our previous study was that the smoothness settings roughly followed a U-shape when plotted against the light spread values of the test. In the present study, however, the shapes of these curves are quite heterogeneous and in most of the cases the curves are far from regaining their initial level. A closer look at the individual data sets of our present matching experiment suggests that there are at least two groups of subjects who show clear differences in their data trends (compare this with the findings from Leloup, Pointer, Dutré, & Hanselaer, 2012, and those from Hansmann-Roth, Pont, & Mamassian, 2017, who could also identify two different response groups among the subjects in their studies on gloss perception): Two of the seven subjects produced data curves that are in good agreement with those from our former study—and this is true for almost all shape and test smoothness conditions (see the black solid curves in Figure B1 in Appendix B; except for the “bunny” condition under the lowest test smoothness level, all curves show the characteristic U-shape). In contrast, the curves of the other five subjects generally lack an increase at higher light spread values (see the black dashed curves in Figure B1 in Appendix B), where the exact shape of the curve also seems to depend on the test object’s shape: Under the shape conditions “blob#1” and “bunny,” the curves can be described as strictly decreasing functions of the light spread, while under the shapes “blob#2” and “statue,” the mean smoothness settings rather seem to reach an asymptote at higher light spread levels.

In this context, our rating data show that with increasing light spread, the satisfaction with the quality of the match systematically decreases and that, especially for higher α values, a perfect match could not be achieved (see Figure 7). This could mean that there was some leeway for finding the “second-best” match, where then different individual preferences may have played a role. These individual preferences in turn may be due to the use of different weights for the set of image cues on which the glossiness estimate is based. In the field of gloss perception, it has repeatedly been found that different sources of information are taken differently into account by different observers (Leloup et al., 2012; Phillips, Ferwerda, & Nunziata, 2010; Wendt et al., 2010; see also Chadwick & Kentridge, 2015).

However, this assumption does not explain why the subjects generally showed the same trend for those conditions where the test objects were presented *dynamically* and under white light: Although the rating values were often similar to those of the corresponding static condition, almost all of the respective matching curves actually had a U-shape (see the solid black curves in Figure 5).

Another factor that could have influenced the smoothness settings of the subjects in the present study is that all experimental conditions were tested in the same session (apart from those that were tested in Experiment 2): In our former study, the test objects were always static and illuminated by achromatic light whereas here the availability of color and motion information changed from trial to trial. This latter procedure may have had an impact on the internal glossiness scale of the subjects, such that, for instance, higher glossiness values would have generally been reserved mostly for colored or dynamic stimuli.

## Conclusions

The highlights produced by different light sources may overlap in the input image and it was found in a previous study that this can have a negative effect on gloss constancy. In the present paper, we investigated whether color and motion information can be used to separate the influence of different light sources and to increase constancy. Our results suggest that the color of the light sources can indeed be used to this end: If the light sources were of clearly different color, gloss constancy performance was much better than when all light sources had the same color. This suggests that gloss perception is not “color blind,” but that it can take advantage of light source color to group highlights according to their cause. The motion information that was available in our experiment was apparently not used in this way. Although motion information slightly increased perceived glossiness overall, it did not help to improve gloss constancy with respect to our light spread variable. We discussed several possible reasons for this negative result. It seems plausible that there are other highlight features besides color that may also be used as grouping factors to separate the influence of different light sources. The approach used in the present experiment seems well suited to investigate whether potential grouping factors are actually used by the visual system.

## Supplemental Material

sj-vid-1-ipe-10.1177 2041669518803964 - Supplemental material for Can Color and Motion Information Be Used to Disentangle the Influence of Multiple Light Sources on Gloss Perception? Click here for additional data file. Supplemental material, sj-vid-1-ipe-10.1177 2041669518803964 for Can Color and Motion Information Be Used to Disentangle the Influence of Multiple Light Sources on Gloss Perception? by Gunnar Wendt and Franz Faul in i-Perception

## Appendix A: ANOVA Results From Experiment 1

**Table A1.** Results of the Five-Way ANOVA Performed on the Matching Data of Experiment 1

Source	Sum of the squares	*df*	Mean of the squares	*F*	*p*
Shape	8.457	3	2.819	675.76	<.001
Smoothness	44.696	2	22.3481	5,357.23	<.001
Lightspread	3.138	6	0.523	125.38	<.001
Color	5.258	1	5.2584	1,260.54	<.001
Motion	1.595	1	1.5953	382.41	<.001
Shape × Smoothness	0.224	6	0.0373	8.93	<.001
Shape × Lightspread	0.462	18	0.0257	6.16	<.001
Shape × Color	0.116	3	0.0388	9.3	<.001
Shape × Motion	0.419	3	0.1396	33.46	<.001
Smoothness × Lightspread	0.458	12	0.0381	9.14	<.001
Smoothness × Color	0.0067	2	0.0034	0.81	.4463
Smoothness × Motion	0.0615	2	0.0307	7.37	<.001
Lightspread × Color	1.379	6	0.2298	55.08	<.001
Lightspread × Motion	0.421	6	0.0702	16.83	<.001
Color × Motion	0.0587	1	0.0587	14.06	<.001
Error	29.13	6,983	0.0042		
Total	95.88	7,055			

*Note.* The table presents the results of a five-way ANOVA performed on the matching data of Experiment 1 with the experimental factors object shape (“Shape”), test smoothness (“Smoothness”), the lightspread parameter α (“Lightspread”), the availability of color information due to different colors for the three point light sources (“Color”), and the availability of motion information due to rotation of the test object (“Motion”). Shown are the results for the five different main effects as well as all first-order interaction effects.

## Appendix B: Two Groups of Subjects Showing Different Data Trends in One Experimental Condition

We found different data trends in perceived glossiness between our previous study (Wendt & Faul, 2017), where the stimuli were presented statically and under white light, and the corresponding condition of our present study: While in the former study the smoothness settings roughly followed a U-shape when plotted against the light spread levels, the curves rather show a further decrease or a flattening at higher light spread values in the present matching experiment (see the dashed black curves in Figure 5). The visual inspection of the individual data sets revealed that the subjects can be roughly divided into two groups which show different data trends under this condition. One group, which consists of two subjects, is in accordance with the trend we found in our former study, that is, the characteristic U-shape is preserved under almost all combinations between test smoothness level and shape condition (see the black solid lines in Figure B1 as well as the red and yellow curves for the two individual subjects belonging to this group). The other group of five subjects, however, does generally not show any increase of the curves at higher light spread values (see the dashed black curves in Figure B1 for the mean curves and the colored curves in the blue-green spectrum for the respective individual curves). Possible reasons for the differences in the shape of the curves between these two groups are discussed in the Discussion section.

## References

[bibr1-2041669518803964] Ahlström U. (1995) Perceptual unit formation in simple motion patterns. Scandinavian Journal of Psychology 36: 343–354. doi:10.1111/j.1467-9450.1995.tb00992.x. 8533053 10.1111/j.1467-9450.1995.tb00992.x

[bibr2-2041669518803964] Angelopoulou E. Poger S. (2003) Color of specular highlights. Proceedings of the SPIE 5007: 298–309. doi:10.1117/12.473905.

[bibr3-2041669518803964] Beck J. Prazdny S. (1981) Highlights and the perception of glossiness. Perception & Psychophysics 30: 407–410. doi:10.3758/BF03206160. 7322822 10.3758/bf03206160

[bibr4-2041669518803964] Börjesson E. Ahlström U. (1993) Motion structure in five-dot patterns as a determinant of perceptual grouping. Perception & Psychophysics 53: 2–12. doi:10.3758/BF03211710. 8433902 10.3758/bf03211710

[bibr5-2041669518803964] Brooks, J. L. (2015). Traditional and new principles of perceptual grouping. In J. Wagemans (Ed.), *The Oxford handbook of perceptual organization* (pp. 57–87). Oxford, England: Oxford University Press.

[bibr6-2041669518803964] Chadwick A. C. Kentridge R. W. (2015) The perception of gloss: A review. Vision Research 109: 221–235. doi:10.1016/j.visres.2014.10.026. 25448119 10.1016/j.visres.2014.10.026

[bibr7-2041669518803964] Cook R. L. Torrance K. E. (1982) A reflectance model for computer graphics. ACM Transactions on Graphics 1: 7–24. doi:10.1145/357290.357293.

[bibr8-2041669518803964] Doerschner K. Fleming R. W. Yilmaz O. Schrater P. R. Hartung B. Kersten D. (2011) Visual motion and the perception of surface material. Current Biology 21: 2010–2016. doi:10.1016/j.cub.2011.10.036. 22119529 10.1016/j.cub.2011.10.036 PMC3246380

[bibr9-2041669518803964] Fleming R. W. Dror R. O. Adelson E. H. (2003) Real-world illumination and the perception of surface reflectance properties. Journal of Vision 35: 347–368. doi:10:1167/3.5.3. 10.1167/3.5.3 12875632

[bibr10-2041669518803964] Forbus, K. (1977). *Light source effects* (Massachusetts Institute of Technology Artificial Intelligence Laboratory Memo No. 422). Retrieved from https://dspace.mit.edu/handle/1721.1/6280.

[bibr11-2041669518803964] Hanada M. (2012) Difference between highlight and object colors enhances glossiness. Perceptual and Motor Skills 114: 735–747. doi:/10.2466/24.27.PMS.114.3.735-747. 22913016 10.2466/24.27.PMS.114.3.735-747

[bibr12-2041669518803964] Hansmann-Roth S. Pont S. C. Mamassian P. (2017) Contextual effects on real bicolored glossy surfaces. Journal of Vision 17: 17 doi:10.1167/17.2.17. 10.1167/17.2.17 28245503

[bibr13-2041669518803964] Hartung B. Kersten D. (2002) Distinguishing shiny from matte. Journal of Vision 2: 551 doi:10.1167/2.7.551.

[bibr14-2041669518803964] Ishihara S. (1967) Test for color blindness, Tokyo, Japan: Kanehara Schuppan Co.

[bibr15-2041669518803964] Kim J. Marlow P. J. Anderson B. L. (2012) The dark side of gloss. Nature neuroscience 15: 1590–1595. doi:10.1038/nn.3221. 23001059 10.1038/nn.3221

[bibr16-2041669518803964] Kim J. Tan K. Chowdhury N. S. (2016) Image Statistics and the Fine Lines of Material Perception. i-Perception 7(4): 1–11. doi:10.1177/2041669516658047. 10.1177/2041669516658047 PMC5030751 27698976

[bibr17-2041669518803964] Klinker G. J. Shafer S. A. Kanade T. (1988) The measurement of highlights in color images. International Journal of Computer Vision 2: 7–32. doi:10.1007/BF00836279.

[bibr18-2041669518803964] Klinker G. J. Shafer S. A. Kanade T. (1990) A physical approach to color image understanding. International Journal of Computer Vision 4: 7–38. doi:10.1007/BF00137441.

[bibr19-2041669518803964] Kooima R. (2008) Generalized perspective projection *(Technical report)*, Baton Rouge: Louisiana State University.

[bibr20-2041669518803964] Lee H. (1986) Method for computing the scene-illuminant chromaticity from specular highlights. Journal of the Optical Society of America A 3: 1694–1699. doi:10.1364/JOSAA.3.001694. 10.1364/josaa.3.001694 3772631

[bibr21-2041669518803964] Lee R. J. Smithson H. E. (2017) Motion of glossy objects does not promote separation of lighting and surface colour. Royal Society Open Science 4: 171290 doi:10.1098/rsos.171290. 29291113 10.1098/rsos.171290 PMC5717688

[bibr22-2041669518803964] Leloup F. B. Pointer M. R. Dutré P. Hanselaer P. (2012) Overall gloss evaluation in the presence of multiple cues to surface glossiness. Journal of Optical Society of America 29: 1105–1114. doi:10.1364/JOSAA.29.001105. 10.1364/JOSAA.29.001105 22673442

[bibr23-2041669518803964] Marlow P. J. Anderson B. L. (2013) Generative constraints on image cues for perceived gloss. Journal of Vision 13: pii: 2 doi:10.1167/13.14.2. 10.1167/13.14.2 24297776

[bibr24-2041669518803964] Marlow P. J. Kim J. Anderson B. L. (2012) The perception and misperception of specular surface reflectance. Current Biology 22: 1909–1913. doi:10.1016/j.cub.2012.08.009. 22959347 10.1016/j.cub.2012.08.009

[bibr25-2041669518803964] Motoyoshi I. Matoba H. (2012) Variability in constancy of the perceived surface reflectance across different illumination statistics. Vision Research 53: 30–39. doi:10.1016/j.visres.2011.11.010. 22138530 10.1016/j.visres.2011.11.010

[bibr26-2041669518803964] Motoyoshi I. Nishida S. Sharan L. Adelson E. H. (2007) Image statistics and the perception of surface qualities. Nature 446: 206–209. doi:10.1038/nature05724. 10.1038/nature05724 17443193

[bibr27-2041669518803964] Ngan, A., Durand, F., & Matusik, W. (2005). *Experimental analysis of BRDF models*. Paper presented at the Proceedings of the Sixteenth Eurographics conference on Rendering Techniques, June 29–July 01, 2005, Konstanz, Germany. doi:10.2312/EGWR/EGSR05/117-126.

[bibr28-2041669518803964] Nishida S. Shinya M. (1998) Use of image-based information in judgments of surface-reflectance properties. Journal of the Optical Society of America A 15: 2951–2965. doi:10.1364/JOSAA.15.002951. 10.1364/josaa.15.002951 9857525

[bibr29-2041669518803964] Olkkonen M. Brainard D. H. (2010) Perceived glossiness and lightness under real-world illumination. Journal of Vision 10: 5 doi:10.1167/10.9.5. 10.1167/10.9.5 PMC2981171 20884603

[bibr30-2041669518803964] Olkkonen M. Brainard D. H. (2011) Joint effects of illumination geometry and object shape in the perception of surface reflectance. i-Perception 2: 1014–1034. doi:10.1068/i0480. 23145259 10.1068/i0480 PMC3485792

[bibr31-2041669518803964] Phillips J. B. Ferwerda J. A. Nunziata A. (2010) Gloss discrimination and eye movement. Proceedings of the SPIE 7527*,*. 75270Z. doi:10.1117/12.845399.

[bibr32-2041669518803964] Phong B. T. (1975) Illumination for computer generated pictures. Communications of the ACM 18: 311–317. doi:10.1145/360825.360839.

[bibr33-2041669518803964] Pont S. C. te Pas S. F. (2006) Material-illumination ambiguities and the perception of solid objects. Perception 35: 1331–1360. doi:10.1068/p5440. 17214380 10.1068/p5440

[bibr34-2041669518803964] Qi L. Chantler M. J. Siebert J. P. Dong J. (2014) Why do rough surfaces appear glossy? Journal of the Optical Society of America. A, Optics, Image Science, and Vision 31: 935–943. doi:10.1364/JOSAA.31.000935. 10.1364/JOSAA.31.000935 24979624

[bibr35-2041669518803964] Qi L. Chantler M. J. Siebert J. P. Dong J. (2015) The joint effect of mesoscale and microscale roughness on perceived gloss. Vision Research 115: 209–217. doi:10.1016/j.visres.2015.04.014. 25969141 10.1016/j.visres.2015.04.014

[bibr36-2041669518803964] Sakano Y. Ando H. (2010) Effects of head motion and stereo viewing on perceived glossiness. Journal of Vision 10: 15 doi:10.1167/10.9.15. 10.1167/10.9.15 21106677

[bibr37-2041669518803964] Sève R. (1993) Problems connected with the concept of gloss. Color Research and Application 18: 241–252. doi:10.1002/col.5080180407.

[bibr38-2041669518803964] Shafer S. A. (1985) Using color to separate reflection components. Color Research and Application 10: 210–218. doi:10.1002/col.5080100409.

[bibr39-2041669518803964] te Pas S. F. Pont S. C. Dalmaijer E. S. Hooge I. T. (2017) Perception of object illumination depends of highlights and shadows, not shading. Journal of Vision 17: 1–15. doi:10.1167/17.8.2. 10.1167/17.8.2 28672368

[bibr40-2041669518803964] Todd J. T. Norman J. F. (2018) The visual perception of metal. Journal of Vision 18: 9 doi:10.1167/18.3.9. 10.1167/18.3.9 29677326

[bibr41-2041669518803964] Tominaga S. Tanaka N. (2000) Estimating reflectance parameters from a single color image. IEEE Computer Graphics & Applications 20: 58–66. doi:10.1109/38.865881.

[bibr42-2041669518803964] Van Assen J. J. R. Wijntjes M. W. A. Pont S. C. (2016) Highlight shapes and perception of gloss for real and photographed objects. Journal of Vision 16: 6 doi:10.1167/16.6.6. 10.1167/16.6.6 27271808

[bibr43-2041669518803964] Vangorp P. Laurijssen J. Dutré P. (2007) The influence of shape on the perception of material reflectance. ACM Transactions on Graphics 26: 77 doi:10.1145/1275808.1276473.

[bibr44-2041669518803964] Wendt G. Faul F. (2017) Increasing the complexity of the illumination may reduce gloss constancy. i-Perception 8(6): 1–40. doi:10.1177/2041669517740369. 10.1177/2041669517740369 PMC5726623 29250308

[bibr45-2041669518803964] Wendt G. Faul F. Ekroll V. Mausfeld R. (2010) Disparity, motion, and color information improve gloss constancy performance. Journal of Vision 10: 7 doi:10.1167/10.9.7. 10.1167/10.9.7 20884605

[bibr46-2041669518803964] Wijntjes M. W. A. Pont S. C. (2010) Illusory gloss on Lambertian surfaces. Journal of Vision 10: 13 doi:10.1167/10.9.13. 10.1167/10.9.13 21106675

